# Human Apurinic/Apyrimidinic Endonuclease (APE1) Is Acetylated at DNA Damage Sites in Chromatin, and Acetylation Modulates Its DNA Repair Activity

**DOI:** 10.1128/MCB.00401-16

**Published:** 2017-03-01

**Authors:** Shrabasti Roychoudhury, Somsubhra Nath, Heyu Song, Muralidhar L. Hegde, Larry J. Bellot, Anil K. Mantha, Shiladitya Sengupta, Sutapa Ray, Amarnath Natarajan, Kishor K. Bhakat

**Affiliations:** a Department of Genetics, Cell Biology and Anatomy, University of Nebraska Medical Center, Omaha, Nebraska, USA; b Department of Radiation Oncology, Houston Methodist Research Institute, Houston, Texas, USA; c Nikon Instrument Inc., Memphis, Tennessee, USA; d Central University of Punjab, Bathinda, India; e Department of Pediatrics, Hematology/Oncology Division, University of Nebraska Medical Center, Omaha, Nebraska, USA; f Eppley Institute for Research in Cancer, University of Nebraska Medical Center, Omaha, Nebraska, USA; g Fred and Pamela Buffet Cancer Center, University of Nebraska Medical Center, Omaha, Nebraska, USA

**Keywords:** APE1, AP site, acetylation, base excision repair, endogenous DNA damage, DNA damage

## Abstract

Apurinic/apyrimidinic (AP) sites, the most frequently formed DNA lesions in the genome, inhibit transcription and block replication. The primary enzyme that repairs AP sites in mammalian cells is the AP endonuclease (APE1), which functions through the base excision repair (BER) pathway. Although the mechanism by which APE1 repairs AP sites *in vitro* has been extensively investigated, it is largely unknown how APE1 repairs AP sites in cells. Here, we show that APE1 is acetylated (AcAPE1) after binding to the AP sites in chromatin and that AcAPE1 is exclusively present on chromatin throughout the cell cycle. Positive charges of acetylable lysine residues in the N-terminal domain of APE1 are essential for chromatin association. Acetylation-mediated neutralization of the positive charges of the lysine residues in the N-terminal domain of APE1 induces a conformational change; this in turn enhances the AP endonuclease activity of APE1. In the absence of APE1 acetylation, cells accumulated AP sites in the genome and showed higher sensitivity to DNA-damaging agents. Thus, mammalian cells, unlike Saccharomyces cerevisiae or Escherichia coli cells, require acetylation of APE1 for the efficient repair of AP sites and base damage in the genome. Our study reveals that APE1 acetylation is an integral part of the BER pathway for maintaining genomic integrity.

## INTRODUCTION

Common forms of DNA damage in the genome are apurinic/apyrimidinic (AP) sites (1, 2). AP sites can be generated either spontaneously through water-mediated depurination or depyrimidination or after removal of oxidized and modified bases by DNA glycosylases (2). Thousands of such AP sites are generated daily in the genome of a human cell (1). These noninstructional AP sites are mutagenic and can inhibit DNA replication and transcription (3, 4). The primary enzyme that repairs AP sites in mammalian cells is the AP endonuclease (APE1), which functions through the base excision repair (BER) pathway (5, 6). Human APE1 is a ubiquitous and multifunctional protein (5). It was originally discovered as a DNA repair enzyme playing a central role in the repair of spontaneously generated AP sites and oxidative and alkylated DNA damage in the genome via the BER pathway (6–8). Apart from its DNA repair function, APE1 functions as a redox activator of many transcription factors (TFs), as well as a direct transcriptional coregulator of many genes (9, 10).

APE1 is essential for embryonic development and for cell viability and/or proliferation in cultures (11–13). Unlike its Escherichia coli prototype, Xth, human APE1 is unique in that it has an N-terminal disordered 42 amino acids (aa) and has both DNA repair and transcriptional regulatory activities (10). In previous studies, we discovered that APE1 can be acetylated (AcAPE1) at lysine 6 (Lys6) and Lys7 residues in the N-terminal domain and that acetylation modulates the transcriptional coregulatory activity of APE1 (14, 15). Moreover, Tell and colleagues, in collaboration with us, found that other Lys residues (Lys27, Lys31, Lys32, and Lys35) in the N-terminal domain of APE1 can be modified by acetylation and these Lys residues modulate the nucleolar localization and BER activity of APE1 (16). We have recently shown that tumor tissue of diverse cancer types has elevated levels of AcAPE1 (17). APE1 was also shown to be ubiquitinated at the Lys24, Lys25, and Lys27 residues (18). Further, using conditional APE1-nullizygous mouse embryo fibroblasts (MEF), we showed that acetylable Lys6 and Lys7 residues of APE1 are essential for cell survival (13). The acetylation sites are conserved in most mammalian APE1 enzymes (10), suggesting that evolutionary conservation or neutralization of the basicity of these Lys residues by acetylation in the N-terminal domain has essential biological functions. Over the last 20 years, the mechanisms by which AP sites are repaired by APE1 *in vitro* via the BER pathway have been extensively investigated (19–23). However, it is largely unknown how APE1 repairs AP sites in mammalian cells.

In this study, we show that APE1 is acetylated after binding to the AP sites in the chromatin and that AcAPE1 is exclusively associated with chromatin throughout the cell cycle. Further, our study revealed the key role of the positive charges of the acetylable Lys residues for the nuclear localization of APE1 and its binding to chromatin. APE1 acetylation induces a conformational change in APE1 which enhances the AP endonuclease activity of APE1 and its interaction with downstream BER proteins. Our study shows that acetylation of APE1 plays a crucial role in the repair of AP sites and oxidative and alkylated base damage in the genome and thus promotes cell survival and proliferation.

## RESULTS

### AcAPE1 is exclusively associated with chromatin throughout the cell cycle.

**FIG 1:**
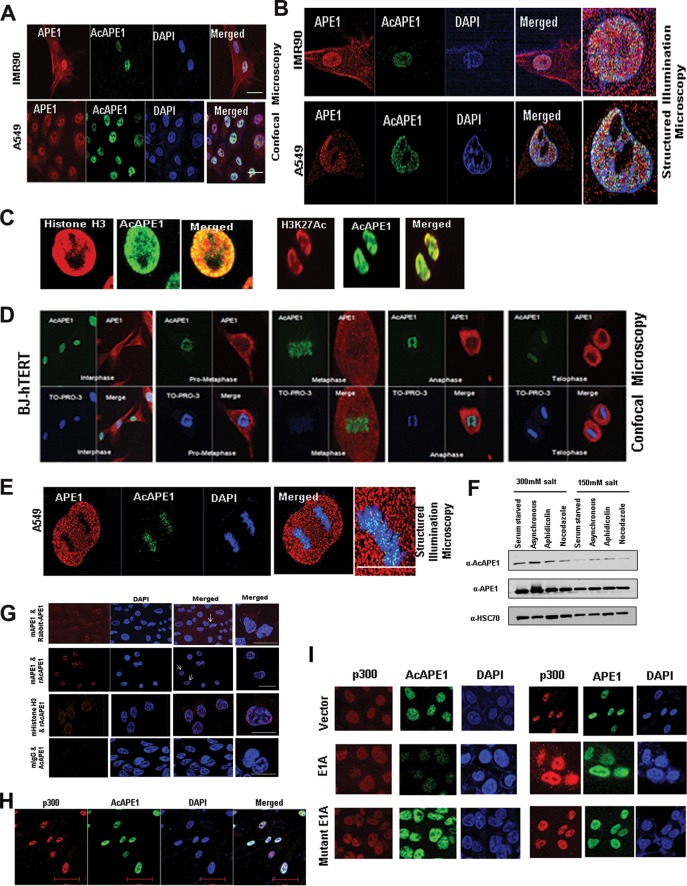
AcAPE1 is exclusively associated with chromatin and remains bound to the condensed chromosomes. (A and B) Asynchronous normal lung fibroblast IMR90 cells and lung adenocarcinoma A549 cells were immunostained with anti-APE1 and anti-AcAPE1 Abs, counterstained with DAPI, and visualized by confocal microscopy and 3D SIM. (C) Colocalization of AcAPE1 with histone H3 or active enhancer-specific histone marker acetylated H3K27 (H3K27Ac). (D) BJ-hTERT cells were serum starved for 72 h and then fixed at different time points. Cells were immunostained with anti-APE1 and anti-AcAPE1 Abs and counterstained with anti-TO-PRO-3 iodide Ab. (E) Mitotic A549 cells were immunostained with anti-APE1 and anti-AcAPE1 and visualized by 3D SIM. (F) BJ-hTERT cells were either serum starved for 72 h (G_0_/G_1_ phase), treated with nocodazole (mitotic cells) or aphidicolin (G_1_/S phase synchronized cells), or untreated, and whole-cell extracts were isolated using 150 mM or 300 mM salt-containing lysis buffer. Western blot analysis for anti-APE1 and anti-AcAPE1 levels was performed. Anti-HSC70 was used as loading control. (G) A proximal ligation assay was performed with mouse anti-APE1 and rabbit anti-APE1 (mAPE1 & Rabbit-APE1), mouse anti-mouse APE1 and rabbit anti-AcAPE1 (mAPE1 & rAcAPE1), and rabbit anti-AcAPE1 and mouse anti-histone H3 (mHistone H3 & rAcAPE1) to confirm the chromatin association of AcAPE1. Mouse IgG (mIgG) and rabbit anti-AcAPE1 were used as a control. At least 50 cells were counted for PLA foci. (H) Colocalization of p300 and AcAPE1 on chromatin (DAPI). (I) HCT116 cells were transfected with E1A and mutant E1A, and at 48 h after transfection, IF was performed. Cells were immunostained with anti-p300 and anti-APE1 or anti-AcAPE1 and counterstained with DAPI.

We investigated the subcellular localization of AcAPE1 using our previously characterized AcAPE1 antibody (Ab) (15, 24). We showed earlier that this AcAPE1 Ab is highly specific for recognizing APE1 species acetylated at the N-terminal Lys6 residue and does not cross-react with a 50-fold excess of unmodified APE1 (24). Moreover, this Ab was unable to recognize ectopic APE1 molecules with mutated Lys6 residues (10). Confocal microscopy and superresolution (110-nm) three-dimensional (3D) structured illumination microscopy (SIM) data revealed AcAPE1 staining to be strictly nuclear, whereas unmodified APE1 was observed both in the nucleus and in the cytoplasm in human normal lung fibroblast (IMR90) cells, human telomerase reverse transcriptase (hTERT)-transformed diploid BJ fibroblast cells (BJ-hTERT cells), as well as human lung adenocarcinoma A549 cells (Fig. 1A, B, and D). Using a chromatin marker histone H3 Ab or an active enhancer marker acetylated H3K27 Ab, we found that AcAPE1 is present on chromatin (Fig. 1C). Furthermore, SIM revealed that AcAPE1 is exclusively localized in the chromatin (Fig. 1B). As chromatin can be easily observed during cell division in mitosis, we examined AcAPE1 localization in mitotic cells. AcAPE1 was found to be exclusively localized to the condensed chromatin at all stages of mitosis, from prometaphase to telophase, both in fibroblast cells and in cancer cells (Fig. 1D and E). The exclusive association of AcAPE1 with chromatin was also confirmed by a proximal ligation assay (PLA) using APE1 or histone H3 and AcAPE1 Abs (Fig. 1G). Our data show that a higher PLA signal localized on DAPI (4′,6-diamidino-2-phenylindole). Consistent with this finding, biochemical extraction of proteins with different salt concentrations demonstrated a higher proportion of AcAPE1 in high-salt fractions at different stages of cell cycles (Fig. 1F). We demonstrated earlier that p300 is the primary acetyltransferase for the acetylation of APE1 (15). We observed that AcAPE1 colocalizes with p300 only on chromatin (Fig. 1H). Furthermore, overexpression of E1A12S, which was shown to bind p300 and inhibit its histone acetyltransferase (HAT) activity (25), significantly reduced the level of AcAPE1 staining but not that of APE1 staining in cells (Fig. 1I), further confirming the specificity of our AcAPE1 Ab. Overexpression of E1A deletion mutant 2-36, which cannot bind to p300, had no effect on AcAPE1 staining (Fig. 1I). Together these data suggest that APE1 is acetylated by p300 and that AcAPE1 is exclusively associated with chromatin in cells.

### Positive charges of acetylable Lys residues but not their acetylation are essential for the chromatin binding of APE1.

**FIG 2:**
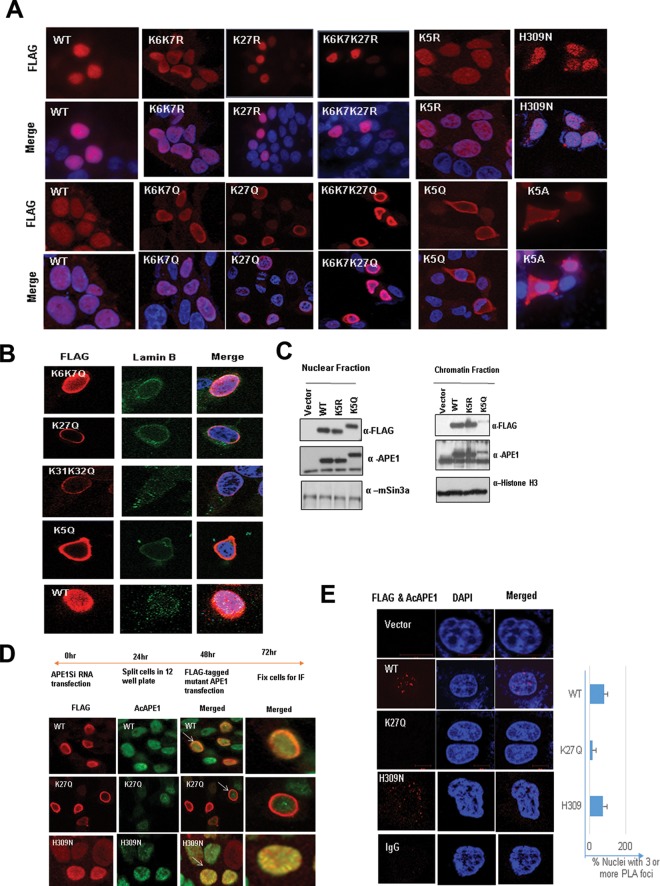
Positive charges of acetylable Lys residues but not their acetylation are essential for chromatin binding of APE1. (A) Cells expressing different mutants with Lys site-specific APE1 mutations were immunostained with anti-FLAG Ab (rows labeled FLAG) and counterstained with DAPI (rows labeled Merge). (B) The subcellular localization of acetylation site mutants was analyzed by immunostaining; cells expressing mutants with different Lys site-specific APE1 mutations were stained with anti-FLAG Ab or anti-lamin B Ab and counterstained with DAPI. (C) Western blot analysis of soluble nuclear and chromatin extracts for FLAG-tagged WT and mutant APE1 levels in cells ectopically expressing these proteins with anti-FLAG. Anti-APE1, anti-histone H3, and anti-mouse Sin3a (mSin3a) were used as controls. (D and E) Schematic overview of the experiment. APE1 was downregulated in HEK293T cells using APE1-specific siRNA (APE1Si RNA), and after 48 h, the cells were transfected with expression plasmids containing WT APE1 or K27Q or H309N mutant APE1. IF was performed using anti-FLAG and anti-AcAPE1 antibodies to check colocalization. At least 50 FLAG-positive cells were counted for colocalization. Cells indicated with arrows were further magnified in the “Merged” column. (E) (Left) PLA was performed using anti-FLAG and anti-AcAPE1 to examine the acetylation of WT APE1 and K27Q and H309N mutant APE1 in cells. (Right) At least 50 cells were counted, and the percentage of the PLA signal was plotted for each APE1 mutant.

To test if the acetylation of APE1 is essential for the association with chromatin, we generated several site-specific acetylable Lys mutants and performed localization studies using immunofluorescence (Fig. 2A) and biochemical fractionation assays (Fig. 2C). We found that mutation of the following to nonacetylable arginine, which maintains a positive charge but cannot be acetylated, did not affect the chromatin association of APE1: Lys6 and Lys7; Lys27 alone; Lys6, Lys7, and Lys27; or all five acetylable Lys residues (Lys6, Lys7, Lys27, Lys31, and Lys32 [K5R]) (Fig. 2A). Surprisingly, neutralization of the positive charges of both Lys6 and Lys7, Lys27 alone, or the three residues Lys6, Lys7, and Lys27 or the mutation of all five acetylable Lys residues to glutamine (K5Q) or alanine (K5A) had a drastic effect on the chromatin binding of APE1 (Fig. 2A) and showed the perinuclear localization of APE1 (Fig. 2B). Our biochemical fractionation assay shows that the neutralization of the positive charges of these Lys residues significantly affected the chromatin association of APE1 (Fig. 2C). We also used an APE1 H309N mutant, which was shown to be catalytically inactive (with no AP endonuclease activity) but can bind to an AP site substrate (26). We found that the H309N mutant could associate with chromatin similarly to wild-type (WT) APE1 (Fig. 2A).

### APE1 is acetylated after binding to AP site damage in the chromatin.

Our confocal and biochemical data clearly indicate that the positive charges of acetylable Lys residues in APE1 but not their acetylation is essential for chromatin association (Fig. 2A and C). Still, we consistently observed that AcAPE1 is exclusively associated with chromatin in both interphase and mitotic cells (Fig. 1B and D). This led us to test whether APE1 is acetylated after binding to AP site lesions in chromatin. If chromatin binding is necessary for APE1 to be acetylated, then the loss of chromatin binding of the K27Q mutant or the K31Q K32Q APE1 mutant (Fig. 2B) is expected to prevent APE1 acetylation at the Lys6 residue in cells and therefore should not be detected by the AcAPE1 Ab. To test this, we downregulated endogenous APE1 levels in HEK293T cells using small interfering RNA (siRNA). After downregulating endogenous APE1, we ectopically expressed FLAG-tagged WT APE1 and K27Q or H309N mutant APE1 and immunostained them with the AcAPE1 Ab to compare the levels of APE1 acetylation at Lys6. We reported earlier that mutation of the Lys27 residue in recombinant APE1 proteins does not affect acetylation by p300 at Lys6 *in vitro* and can be detected by the AcAPE1 Ab by Western blot analysis (16). The results of our immunofluorescence assay (IF) (Fig. 2D) and PLA (Fig. 2E) showed the colocalization of the AcAPE1 and FLAG Abs only in cells expressing FLAG-tagged WT APE1 and H309N mutant APE1 and not in chromatin-binding defective K27Q mutant-expressing cells, providing evidence that a chromatin association is necessary for APE1 to be acetylated.

**FIG 3:**
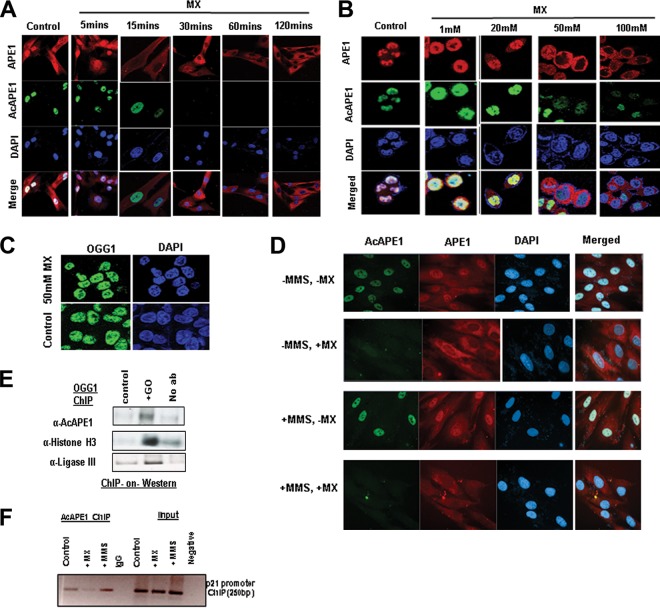
APE1 is acetylated after binding to AP sites in the chromatin. (A) BJ-hTERT cells were treated with MX (50 mM) for the indicated times. IF was performed using anti-APE1 and anti-AcAPE1, and counterstaining with DAPI was used. (B) HCT116 cells were treated with various doses of MX for 30 min, IF was performed using anti-APE1 and anti-AcAPE1, and counterstaining with DAPI was used. (C) HCT116 cells were treated with 50 mM MX for 30 min, IF was performed using anti-OGG1, and counterstaining with DAPI was used. (D) BJ-hTERT cells pretreated with 50 mM MX for 30 min or not pretreated were exposed to MMS (2 mM) for 1 h. IF was performed using anti-APE1 and anti-AcAPE1, and counterstaining with DAPI was used. Confocal microscopy was used to visualize the AcAPE1 levels in control cells and cells treated with MMS or MX, or both. (E) ChIP with anti-OGG1 antibody followed by Western blotting (ChIP-on-Western) was performed to examine the association of AcAPE1 and ligase III on chromatin after induction of DNA damage with GO. (F) The association of AcAPE1 with the endogenous p21 promoter in control or MMS- or MX-treated cells was examined by promoter-directed ChIP using anti-AcAPE1.

To directly test that the acetylation of APE1 occurs after binding to AP site lesions in the genome in cells, we abrogated the binding of APE1 to AP sites by methoxyamine (MX). Several earlier studies have established that MX covalently binds to AP sites to form methoxyamine-bound AP (MX-AP) sites and competitively inhibits the binding of APE1 to AP sites (27–29). These MX-AP sites are resistant to recognition and repair by APE1 (27, 28). Thus, we treated the cells with different doses of MX for different time periods and found that treatment with MX resulted in a dose- and time-dependent inhibition of the chromatin association of endogenous APE1 (Fig. 3A and B). This indicates that the observed chromatin association of APE1 was primarily because AP sites damage binding on the genome (Fig. 3A and B). Interestingly, we observed that MX treatment completely abrogated APE1 acetylation in a dose- and time-dependent manner (Fig. 3A and B). However, MX treatment did not affect the chromatin association of OGG1 (Fig. 3C), a DNA glycosylase which recognizes 8-oxoguanine DNA base damage (30). Thus, MX treatment does not inhibit the chromatin association of the other initial enzyme involved in the BER pathway. To further support the observation that APE1 acetylation occurs after binding to the AP sites, we induced the generation of AP sites in the genome by treatment with the alkylating agent methyl methanesulfonate (MMS) or glucose oxidase (GO), an oxidizing agent that induces oxidative base damage. This oxidative or alkylated base damage subsequently generated AP sites after removal of the oxidized or alkylated bases by DNA glycosylases (31). As shown in Fig. 3D, blocking the AP site with MX treatment abrogated the acetylation of APE1 even after induction of the AP sites with MMS treatment (Fig. 3D). Moreover, our biochemical assay revealed that treatment with GO significantly enhanced the levels of chromatin-bound AcAPE1 (Fig. 3E). We also examined the association of APE1 with the endogenous p21 promoter region in cells via chromatin immunoprecipitation (ChIP) using the AcAPE1 Ab after the induction of DNA damage with MMS treatment. A significant enrichment of an AcAPE1-bound p21 promoter region was observed in MMS-treated cells compared to control cells (Fig. 3F). Similarly, MX treatment reduced the enrichment of the AcAPE1-bound promoter region (Fig. 3F). Together, these data suggest that APE1 is acetylated after binding to AP sites in chromatin in cells.

### Acetylation of APE1 enhances its AP endonuclease activity or catalytic efficiency.

**FIG 4:**
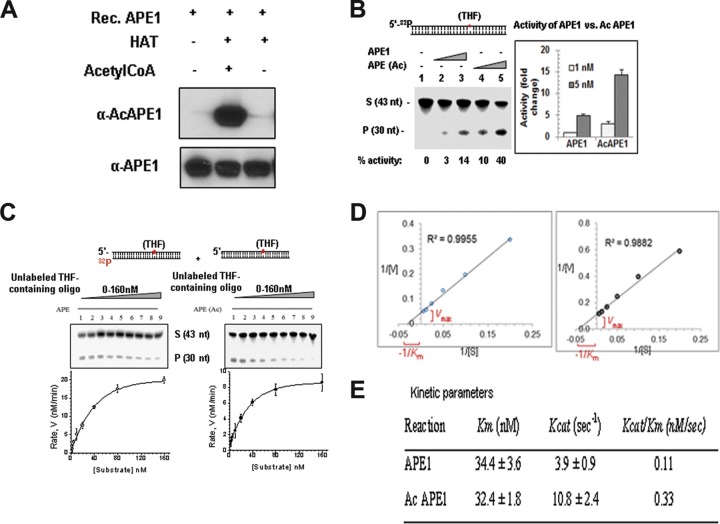
Acetylation of APE1 enhances its AP endonuclease activity. (A) Recombinant (Rec.) APE1 was incubated with the p300 HAT domain either in the presence or the absence of acetyl-CoA, and Western blot analysis was performed with anti-APE1 and anti-AcAPE1 Abs to confirm the acetylation of APE1. (B) Incision of the THF (reduced AP site)-containing 43-mer duplex oligonucleotide (S, substrate) by APE1 and *in vitro*-acetylated APE1. nt, nucleotides; P, the cleaved product. (C and D) The values of the kinetic parameters *K_m_* and *k*_cat_ were calculated by incubating 33 pM enzyme at 37°C for 3 min with substrates at various concentrations (0 to 160 nM). The enzyme kinetics data were fitted into a nonlinear least-squares regression to obtain *V*_max_ and *K_m_* values by use of the Michaelis-Menten equation and SigmaPlot software. (E) Comparison of kinetic parameters between APE1 and AcAPE1.

We investigated directly whether acetylation affects the AP endonuclease activity of APE1 *in vitro*. Purified WT APE1 was incubated with the p300 HAT domain either in the presence or in the absence of acetyl coenzyme A (acetyl-CoA), and then the acetylation of APE1 was confirmed by Western blot analysis using our AcAPE1-specific Ab (Fig. 4A). We found that acetylation of APE1 increased its AP endonuclease activity in a dose-dependent manner (Fig. 4B). We determined the steady-state parameters for both recombinant unmodified APE1 and AcAPE1 during the period of the linear increase (up to 3 min) of product formation (Fig. 4C and D). Enzyme kinetic analysis showed that both APE1 and AcAPE1 have comparable binding affinities (*K_m_* values) for the substrate AP sites (Fig. 4E). However, acetylation enhanced the catalytic turnover (*k*_cat_ value) of APE1 *in vitro* (Fig. 4E). Thus, AcAPE1 has a higher (∼−3-fold) *k*_cat_/*K_m_* ratio, i.e., increased catalytic efficiency.

### APE1 acetylation enhances its interaction with downstream BER proteins and stability on chromatin.

**FIG 5:**
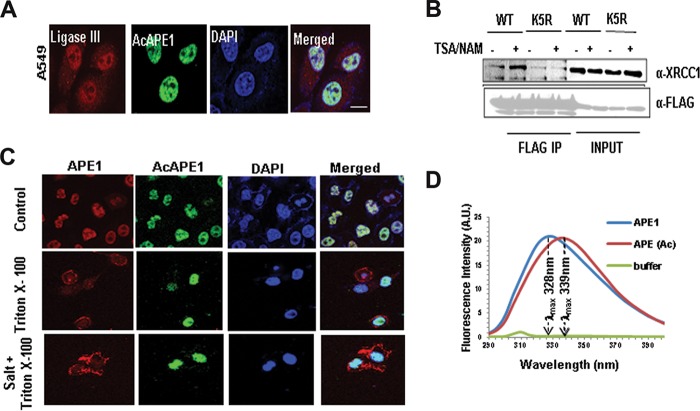
APE1 acetylation enhances its stability on chromatin and its interaction with downstream BER proteins. (A) Colocalization of ligase III and AcAPE1 in A549 cells. Cells were immunostained with anti-ligase III and anti-AcAPE1 Abs. (B) WT or K5R mutant APE1-overexpressing HEK293T cells were treated with TSA-nicotinamide (NAM) for 6 h or not treated, and the nuclear extract was immunoprecipitated using anti-FLAG Ab and immunoblotted with anti-XRCC1 and anti-FLAG Abs. (C) A549 cells were fixed with paraformaldehyde before (top) or after treatment with Triton X-100 (0.5%) (middle) or Triton X-100 plus salt (100 mM KCl) (bottom) and immunostained with anti-APE1 or anti-AcAPE1 Abs and counterstained with DAPI. (D) Acetylation of APE1 induces a conformational change in APE1. The distinct intrinsic fluorescence emission spectra of APE1 and AcAPE1 at 280 nm are shown. A.U., absorbance units.

In cells, the total (complete) repair of AP sites is dependent on not only the AP endonuclease activity of APE1 but also its interaction and coordinated recruitment of downstream BER proteins, such as DNA polymerase beta (Polβ) and XRCC-1/DNA ligase III (32–34). APE1 has been shown to incise the AP site, remains tightly bound to the cleaved AP site product, and serves as a mediator for the next step in the BER pathway. Thus, when it is bound to the cleaved AP site, APE1 physically interacts with Polβ and significantly stimulates its deoxyribose phosphate (dRP) lyase activity (26, 34). Moreover, an interaction of APE1 with XRCC1 was shown (35). Consistent with this idea, we observed that AcAPE1 colocalizes with ligase III in chromatin (Fig. 5A). Furthermore, we found that treatment with trichostatin A (TSA), which enhances APE1 acetylation levels (36), increased the association of XRCC1 with WT APE1 but not with the nonacetylable K5R mutant (Fig. 5B). This indicates that the acetylation of APE1 enhances its interaction with XRCC1 (37). Interestingly, we found that preextraction of loosely associated proteins with 0.5% Triton X-100 and salt prior to fixation significantly decreased the level of staining of nonmodified APE1 but did not have any effect on AcAPE1 staining in the nucleus (Fig. 5C). This suggests that APE1 is more stable on chromatin when it is acetylated.

### Acetylation induces a conformational change in APE1.

So far, our data suggest that upon binding to the AP site in the chromatin, APE1 is acetylated and AcAPE1 has a higher catalytic efficiency. We tested the possibility that the acetylation-mediated neutralization of the positive charges of Lys residues in the N terminus induces a conformational change in APE1 and that this increases its catalytic efficiency. We compared the UV fluorescence of both unmodified APE1 and AcAPE1. WT APE1 contains 7 tryptophan (Trp) residues and 11 tyrosine (Tyr) residues, all of which are located in its globular core domain (aa 42 to 318) (38). The wavelength for the maximal fluorescence (λ_max_) of Trp residues that are exposed to water is about 340 to 350 nm, whereas the λ_max_ of totally buried residues is about 330 nm (39). Excitation at 280 nm (for both Tyr and Trp) showed a typical Trp emission spectrum of WT APE1 with a λ_max_ of 329 nm. However, AcAPE1 showed a red shift (λ_max_, 339 nm), indicating a more solvent-exposed environment for the Trp residues when APE1 is acetylated (Fig. 5D). These data together suggest that acetylation of APE1 induces a conformational change in APE1.

### The absence of acetylation in cells expressing APE1 accumulates AP sites in the genome.

**FIG 6:**
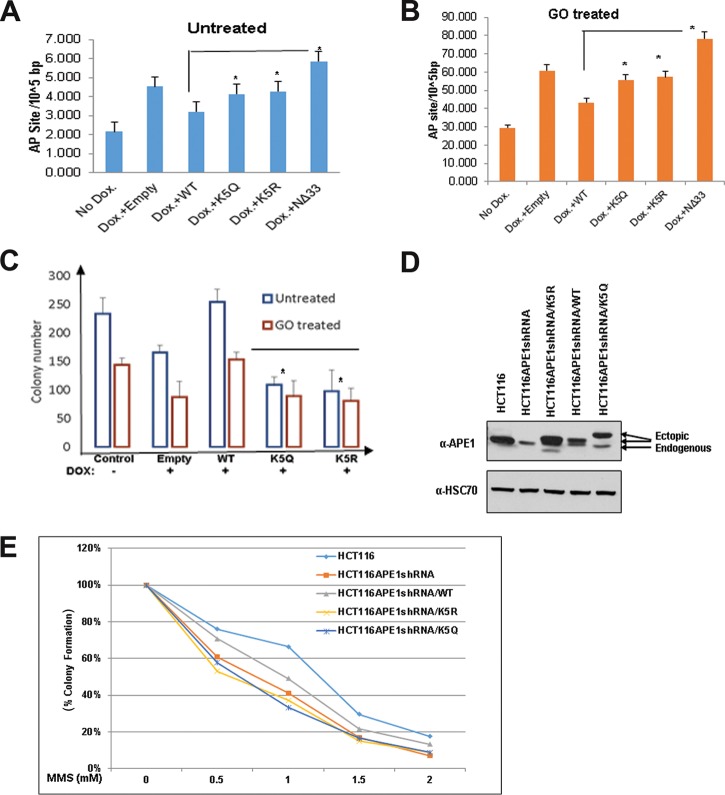
APE1 acetylation is essential for cell survival and/or cell proliferation, and the absence of APE1 acetylation sensitizes cells to DNA-damaging agents. (A and B) Endogenous APE1 was downregulated in HEK293T^APE1siRNA^ cells using Dox treatment. (A) FLAG-tagged WT APE1, acetylation-defective mutants (mutants with the mutation of Lys6, Lys7, Lys27, Lys31, and Lys32 to nonacetylable arginine [K5R] or glutamine [K5Q]), or NΔ33 mutants were further overexpressed in these cells. Cells were treated with glucose oxidase (100 ng/ml for 30 min) or not treated, and the number of AP sites was measured using an ARP kit. (B) Bar diagram representing the number of AP sites/10^5^ bp in the presence of different APE1 mutants compared to that for the vector control. Error bars indicate means ± SDs (*n* = 3). (C) HEK293T^APE1siRNA^ cells were treated with DOX or not treated, and WT APE1 and K5R and K5Q mutant APE1 were ectopically expressed. Cells were treated with glucose oxidase (100 ng/ml for 30 min) or not treated, and a colony formation assay was performed. The bar diagram shows the number of colonies formed in the presence of the different APE1 mutants compared to that for the vector control. Error bars indicate means ± SDs (*n* = 3). (D and E) HCT116 cells constitutively expressing APE1 shRNA (HCT116^APE1shRNA^ cells) were transfected with FLAG-tagged WT APE1 or acetylation-defective APE1 mutants (mutants with mutation of Lys6, Lys7, Lys27, Lys31, and Lys32 to nonacetylable arginine [K5R] or glutamine [K5Q]). (D) Western blot analysis was performed to examine APE1 levels using an anti-APE1 Ab. Anti-HSC70 was used as a loading control. (E) Sensitivity to damage was measured in HCT116^APE1shRNA^ cells ectopically expressing different APE1 mutants. Cells were treated with increasing doses of MMS for 1 h, and a colony formation assay was performed.

The observation that APE1 acetylation enhances its endonuclease activity raises the possibility that in the absence of acetylable Lys residues, cells will accumulate AP sites in the genome. We quantitated the AP sites in the genomes of cells expressing WT APE1 and nonacetylable APE1 mutants by using an aldehyde-reacting probe (ARP). We used HEK293T cells stably expressing APE1-specific siRNA (HEK293T^APE1siRNA^ cells) under the control of a doxycycline (Dox)-inducible promoter, as described earlier (15). We treated the cells with Dox (1 μg/ml) to deplete endogenous APE1 and then ectopically expressed FLAG-tagged WT APE1 or its mutants lacking the acetylation sites. The AP sites in the genome were quantitated using ARP (31, 40). As expected, we observed that the depletion of endogenous APE1 with Dox significantly increased the number of AP sites in the genome compared to that for the control (Fig. 6A). This effect could be partially rescued by ectopic expression of WT APE1 but not by its nonacetylable K5R mutant, the chromatin-binding defective K5Q mutant, or a mutant with an N-terminal deletion of 33 aa (the NΔ33 mutant) (Fig. 6A). We also treated these cells with glucose oxidase to induce oxidative damage, which in turn produced AP sites in the genome. As shown in Fig. 6B, treatment with glucose oxidase significantly enhanced the number of AP sites in the genomes of cells in which APE1 was downregulated. These could be reduced by ectopic expression of WT APE1 but not by either the nonacetylable K5R mutant or the nonacetylable K5Q mutant. Together, these data indicate that the presence of acetylable Lys residues and acetylation of these residues in APE1 play a crucial role in endogenous DNA damage or AP site repair in cells.

### APE1 acetylation plays a role in cell survival and/or proliferation in response to genotoxic stress.

We examined the role of APE1 acetylation in cell survival and/or proliferation, measuring cell survival by a clonogenic survival assay. As expected, we observed that the depletion of endogenous APE1 with Dox decreased the number of viable colonies compared to that for the control (Fig. 6C). This effect could be rescued by ectopic expression of WT APE1 but not by nonacetylable K5R or K5Q mutant APE1 (Fig. 6C). We further examined the role of APE1 acetylation in cell survival or proliferation after the induction of DNA damage. Treatment with GO decreased the number of viable colonies which could be rescued by ectopic expression of WT APE1 but not by its nonacetylable mutants (Fig. 6C). Furthermore, we transfected HCT116 cells that stably express APE1 short hairpin RNA (shRNA) (HCT116^APE1shRNA^ cells) with FLAG-tagged WT APE1 or the nonacetylable K5R or K5Q mutant. Western blot analysis was used to determine the expression levels of WT and mutant APE1 (Fig. 6D). We found that treatment with the alkylating agent MMS sensitized the HCT116^APE1shRNA^ cells in a dose-dependent manner (Fig. 6E). This effect could be partially rescued by ectopic expression of WT APE1 but not by the nonacetylable K5R or K5Q mutant protein, which was present at higher levels in the cells (Fig. 6E and D). Our Western blot analysis showed that the expression levels of ectopic FLAG-tagged WT APE1 were not equal to the level of endogenous APE1 present in control HCT16 cells (Fig. 6D; compare the first and fourth lanes). This may explain why ectopic WT APE1 was not able to fully rescue the sensitivity of HCT116^APE1shRNA^ cells. Nonetheless, this effect could be partially rescued by the ectopic expression of WT APE1 but not by the nonacetylable K5R or K5Q mutant protein, which was present at higher levels in the cells (Fig. 6E and D). These data indicate that APE1 acetylation plays a role in cell survival and/or proliferation and that the absence of APE1 acetylation in cells sensitizes them to DNA-damaging agents.

## DISCUSSION

Unrepaired AP sites in the genome can inhibit transcription and block DNA replication (3, 4, 41). Thus, the presence of an efficient repair mechanism is essential for cell survival and/or proliferation. We reported earlier that multiple Lys residues in the intrinsically disordered N-terminal domain of APE1 are modified by acetylation in cells (14, 16). Further, using conditional APE1-nullizygous mouse embryo fibroblasts (MEF), we showed that both the DNA repair function and the acetylable Lys6 and Lys7 residues of APE1 are essential for cell survival (13). Moreover, a recent study showed that the absence of acetylation at the Lys6 and Lys7 sites in APE1 or its DNA repair function results in telomere fusion and mitotic defects (42). Given the key role of APE1 in the repair of AP sites, which inhibits transcription and replication (2, 3, 41), the essential role of the DNA repair function of APE1 in cell survival is not surprising (7, 12, 13, 43). However, what was unexpected was the critical importance of APE1's acetylation for cell survival and proliferation (13). In this study, we demonstrated that the acetylation of APE1 plays a crucial role during the process of AP site repair in the genome in cells via the BER pathway. Our study documents that APE1 is acetylated after binding to AP sites in the chromatin and that acetylation not only improves the catalytic efficiency of APE1 but also may facilitate the coordination and recruitment of the downstream enzyme in the BER pathway. Thus, the acetylation of APE1 is likely an integral part of the APE1-dependent BER pathway for maintaining DNA integrity.

Although the N-terminal domain (aa 1 to 61) of APE1 is dispensable for its *in vitro* DNA repair activity (44, 45), this study unraveled the novel regulatory role of acetylation of multiple Lys residues in this domain in AP site repair both *in vitro* and in cells. Our study provides direct evidence that after acetylation APE1 enhances its AP endonuclease activity *in vitro*. We have also provided evidence that the absence of acetylation in cells expressing APE1 results in the accumulation of AP sites in the genome and the genome becomes sensitive to DNA-damaging agents. Several lines of evidence support the suggestion that APE1 is acetylated at the Lys6 residue after binding to AP sites in the chromatin. First, AcAPE1 is exclusively present on chromatin throughout cell cycles in all types of cells, including primary, transformed, and tumor cells. Second, the inhibition of binding of APE1 to AP sites by MX treatment abrogates APE1 acetylation in a dose- and time-dependent manner. Third, APE1 mutants that are proficient in acetylation but cannot bind to chromatin cannot be acetylated in cells. Finally, induction of AP sites in the genome by MMS treatment enhanced APE1 acetylation and the occupancy of AcAPE1 on chromatin.

Stimulation of the AP endonuclease activity of APE1 due to acetylation suggests multiple possible mechanisms. An acetylation-induced conformational change in APE1 could either increase its affinity for the substrate AP site in DNA, facilitate AP site cleavage, or decrease APE1's affinity for the product (the cleaved AP site), thus increasing its turnover. Our data showing that both WT APE1 and AcAPE1 have comparable affinities (*K_m_* values) for the substrate AP sites but different *k*_cat_ values suggest that an acetylation-induced conformational change in APE1 could either facilitate AP site cleavage or increase the dissociation of APE1 from the product (the cleaved AP site), thus increasing its turnover. However, APE1 has been shown to incise the AP site, remain tightly bound to the cleaved AP site product, and serve as a mediator for the next step in the BER pathway (34). Efficient complete (total) repair of the AP site is dependent not only on APE1 endonuclease activity but also on the coordination and interaction of APE1 with downstream BER proteins (22, 32, 34, 35). Thus, when bound to the cleaved AP site, APE1 physically interacts with DNA Polβ and significantly stimulates its dRP lyase activity (26, 34). The interaction of APE1 with XRCC1 was also shown (35). Consistent with this idea, we observed that AcAPE1 interacts with ligase III in chromatin and that acetylation of APE1 enhances its interaction with XRCC1. Thus, after acetylation, APE1 not only facilitates AP site cleavage in the chromatin but may also enhance the coordination of total AP site repair in cells.

In the absence of any structural information about full-length APE1 or its binary complex with the AP site containing oligonucleotides, it is not easy to establish if these acetylable Lys residues directly interact with AP sites containing DNA or a C-terminal active-site domain. Our data show that the positive charges of the acetylable Lys residues are important for its nuclear retention and/or chromatin binding and that neutralization of the positive charges of acetylable Lys residues reduce its chromatin association. It is likely that basic Lys residues in the N-terminal domain of APE1 may dynamically interact with DNA or with acidic residues in the C-terminal core domain. Alternatively, these Lys residues may be involved in a direct interaction with partner proteins, including nuclear importin complexes for nuclear localization or nucleosome remodeling complexes that facilitate the binding access of APE1 to AP sites in nucleosomes in the context of chromatin in cells. Consistent with this, earlier studies by our group and others have shown that the APE1 N-terminal 33 aa are required for interaction with many interacting partners and that the neutralization of positive charges in APE1 affects their interaction (15, 46). Intrinsically unstructured regions and electrostatic forces are known to be generally important for intramolecular interactions. It has been shown that NEIL1, a DNA glycosylase involved in oxidative damage repair, has an intrinsically disordered C-terminal domain (aa 311 to 389) that is involved in the intramolecular interaction with a negatively charged core domain (47). This domain was also shown to be essential for the interaction of NEIL1 with many binding partners (48).

**FIG 7:**
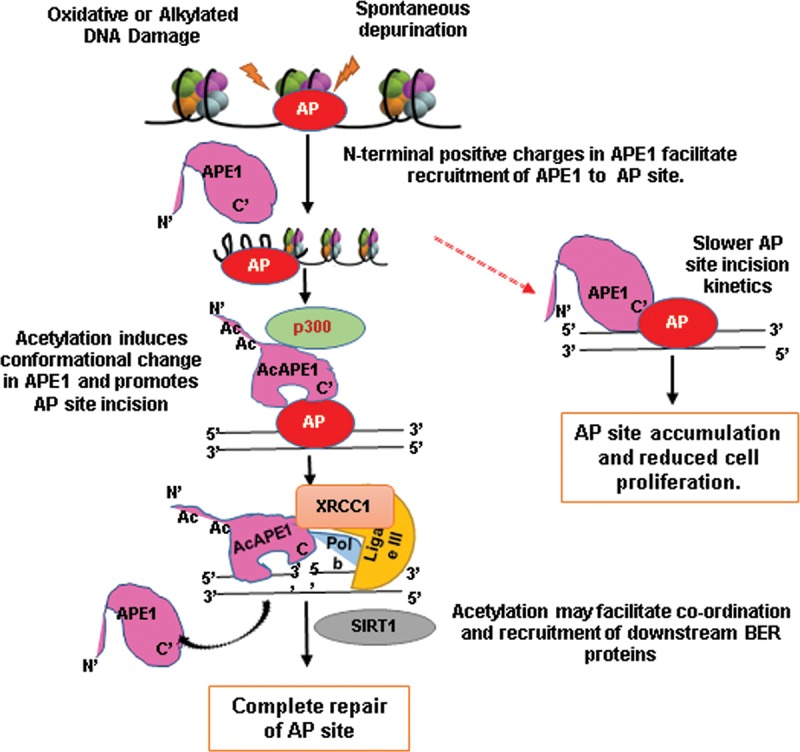
Schematic model for regulation of AP site repair in cells by acetylation of APE1 via the BER pathway.

We propose a model (Fig. 7) by which APE1 acetylation regulates AP site repair in cells. When APE1 locates AP sites in the genome, the first stable APE1-AP DNA complex is formed. Subsequent recruitment of p300 to the damage sites acetylates APE1. Acetylation-mediated neutralization of multiple positive charges in the N-terminal domain induces a conformational change in the catalytically active domain in APE1, which facilitates AP site cleavage in DNA. Consistent with this, one study has shown that the neutralization of the positive charges of the Lys residues in the N-terminal domain or deletion of the N-terminal domain of APE1 significantly enhanced the AP endonuclease activity of APE1 *in vitro* (46). Moreover, our current data show a change (red shift) in the Trp emission in APE1 after its acetylation. Since the acetylation of APE1 improves the rate of APE1 turnover and also enhances the interaction with XRCC1, it appears that the acetylation of APE1 has evolved not only to improve the catalytic efficiency but also to facilitate the coordination and recruitment of the downstream enzyme in the BER pathway. We hypothesize that, after AP site cleavage, AcAPE1 remains bound to the cleaved AP sites and the subsequent recruitment of Polβ displaces APE1 from the 5′-deoxyribose phosphate of the cleaved AP site (but not from DNA), which is enhanced when APE1 is acetylated. Thus, the acetylation-induced conformational change in APE1 not only enhances its cleavage activity but also, at the same time, may facilitate coordination with the next BER proteins. Such a mechanism might also help to explain why AcAPE1 is associated with ligase III and the XRCC1 complex and why acetylation stimulates their interaction in cells. Finally, recruitment of the histone deacetylase SIRT1 (49, 50) deacetylates AcAPE1 and displaces APE1 from the DNA. Consistent with this finding, an earlier study showed SIRT1 can deacetylate APE1 *in vitro* and in cells and that it regulates cellular BER (50).

Our novel discovery highlights that the conserved acetylable Lys residues in the unique N-terminal domain (present only in mammalian APE) of APE1 and that their modifications have evolved to finely regulate and coordinate the efficient repair of the AP site in mammalian cells. Thus, mutation of either catalytically active sites or acetylation sites in the N-terminal domain would have profound cellular consequences, such as growth arrest and/or cell death. Consistent with this hypothesis, we showed earlier that both the DNA repair function and the acetylable Lys6 and Lys7 residues of APE1 are essential for cell survival (13). Moreover, a recent study by Madlener and colleagues showed that APE1 is essential in maintaining telomere length, presumably through maintenance of DNA integrity in that region (42). They found that both the DNA repair function-active H309 or N212 site and the acetylable Lys6 and Lys7 sites in APE1 are essential for maintaining the telomere length (42). We believe that mitotic telomeres might be prone to oxidative damage, which generates AP sites, and that the absence of AcAPE1-mediated AP site repair leads to the accumulation of DNA breaks in this region, which would then lead to telomere fusion and mitotic defects. Indeed, they found that the absence of acetylation of APE1 or its DNA repair function leads to mitotic defects. Of note, our confocal IF data demonstrate the presence of AcAPE1 on condensed chromatin during all stages of mitosis. This indicates that AP sites are also generated in the genome during mitosis; AcAPE1-mediated BER may be also operative. Further studies are necessary to establish this.

Nonetheless, our findings demonstrate that APE1 acetylation is an integral part of the BER pathway in cells for maintaining genomic stability and also provide the mechanism by which APE1 acetylation plays a key role in the repair of AP sites or oxidative DNA damage. Our study also implicates that the dysregulation of the APE1 acetylation/deacetylation cycle may lead to genomic instability and cause many human diseases, including cancer and premature aging.

## MATERIALS AND METHODS

### Cell lines, plasmids, siRNAs, transfection, and treatments.

Human embryonic kidney HEK293T cells (ATCC CRL-1573) and inducible HEK293T^APE1siRNA^ cells in which APE1 was downregulated were cultured in high-glucose Dulbecco modified Eagle medium (DMEM; Thermo Fisher Scientific) with 10% fetal calf serum (FCS; Sigma) and an antibiotic mixture consisting of 100 U/ml penicillin and 100 μg/ml streptomycin (Gibco-BRL) as described previously (15). Human colon cancer HCT116 cells (ATCC CCL-247) were grown in McCoy 5A medium (Thermo Fisher Scientific). The generation of HCT116 cells stably expressing APE1-specific shRNA or control shRNA was described previously (51). hTERT-immortalized human foreskin fibroblast BJ-5ta cells (BJ-hTERT cells; ATCC CR-4001), normal lung fibroblast IMR90 cells (ATCC CCL-186), and lung adenocarcinoma A549 cells (ATCC CCL-185) were cultured in low-glucose DMEM (Thermo Fisher Scientific) with FCS and antibiotics. All cell lines were authenticated by short tandem repeat DNA profiling in August 2015 by Genetica DNA Laboratories, Burlington, NC. Mutation of Lys residues (K6, K7, K27, K31, and K32) singly or in combination to arginine, glutamine, or alanine in FLAG-tagged APE1 in plasmid pCMV5.1 was generated using a site-directed mutagenesis kit (Agilent, Stratagene) following the manufacturer's protocol. Exponentially growing HCT116 cells stably expressing APE1-specific shRNA were transfected with wild-type (WT) APE1 or plasmids expressing the mutant in which K6, K7, K27, K31, and K32 were changed to arginine (K5R) or glutamine (K5Q). In another set of experiments, HEK293T^APE1siRNA^ cells were treated with doxycycline (1 μg/ml) for 5 days to knock down the APE1 levels, and then the cells were transfected with FLAG-tagged WT APE1, K5R or K5Q mutant APE1, or mutant APE1 from which the N-terminal 33 amino acids were deleted (the NΔ33 mutant) as described elsewhere (15, 24, 52, 53). Plasmids expressing adenovirus EIA12S or mutant E1A (with a deletion of amino acids 2 to 36) were described earlier (25). Cells were transfected using the Lipofectamine 2000 reagent (Invitrogen) and harvested after 48 h. Methoxyamine, glucose oxidase, and methyl methanesulfonate were obtained from Sigma.

### Immunofluorescence and PLA.

The different types of cells (HEK293T, HCT116, A549, BJ-hTERT, and IMR90 cells) were grown on coverslips. After transfection with the plasmids indicated above, the cells were fixed with 4% formaldehyde (Sigma) for 20 min and permeabilized with 0.2% Triton X-100 (Sigma) containing blocking solution (goat serum, glycine, sodium azide) for 1 h. Slides were incubated overnight at 4°C with the primary antibodies, washed three times with phosphate-buffered saline (PBS), and incubated with the secondary antibodies Alexa Fluor 488-conjugated anti-mouse IgG (1:500; Life Technologies) or Alexa Fluor 594-conjugated anti-rabbit IgG (1:500; Life Technologies) at room temperature for 1 h. After being washed three times, the slides were mounted on Vectashield-DAPI-containing medium (Vector Laboratories). The primary antibodies used for immunofluorescence studies were mouse monoclonal anti-APE1 (1:100; catalog number NB100-116; Novus Biologicals), anti-AcAPE1 (1:50) (24), anti-FLAG (1:50; catalog number F3165; Sigma), anti-p300 (1:100; catalog number 61401; Activemotif), anti-OGG1 (1:50), anti-histone H3 (1:100; catalog number sc10809; Santa Cruz), anti-H3K27 acetylated histone (1:100; catalog number 05-1334; Millipore), anti-ligase III (1:50; catalog number NB100-152; Novus Biologicals), and anti-lamin B (1:50; catalog number ab16048; Abcam). The proximal ligation assay (PLA) was performed following the manufacturer's protocol (Duolink *in situ* fluorescence PLA technology; Sigma). The numbers of signals for colocalization of these two molecules were calculated using Duolink PLA software. For the quantification of at least 50 cells, nuclei were counted for each experiment, and standard deviations (SDs) from three independent experiments were calculated. Images were acquired by use of a fluorescence microscope with a 63× oil immersion lens (LSM 510; Zeiss), and structured illumination microscopy (SIM) was done with an Elyra PS.1 microscope (Carl Zeiss) by using a 63× objective with a numerical aperture of 1.4. ImageJ software was used to measure Manders colocalization using the JaCoP plug-in.

### Isolation of cytoplasmic, nuclear, and chromatin fractions and Western blot analysis.

Cells were lysed in cytosol extraction buffer (Tris-HCl, 10 mM, pH 8; sucrose, 0.34 mM; CaCl_2_, 3 mM; MgCl_2_, 2 mM; EDTA, 0.1 mM; dithiothreitol [DTT], 1 mM; Nonidet P-40, 0.1%; protease inhibitor cocktail). After centrifugation, the supernatant was collected as the cytoplasmic fraction. The pellet was then dissolved in nuclear extraction buffer (HEPES, 20 mM, pH 7.9; EDTA, 3 mM; glycerol, 10%; potassium acetate, 10 mM; magnesium chloride, 1.5 mM; DTT, 1 mM; Nonidet P-40, 0.5%; protease inhibitor cocktail) and collected as the nuclear fraction. Finally, the pellet was dissolved in chromatin extraction buffer (HEPES, 150 mM; MgCl_2_, 1.5 mM; potassium acetate, 150 mM; glycerol, 10%; protease inhibitor cocktail), 4 U nuclease (DNase and RNase) was added, and the mixture was incubated for 30 min at 37°C. After centrifugation, the supernatant was collected as the chromatin fraction. The whole-cell lysates were prepared with cold lysis buffer containing 50 mM Tris-HCl, pH 7.5, 150 mM NaCl, 1% Triton X-100, 0.1 mM EDTA, and protease inhibitor cocktail buffer tablet (PI; Roche Diagnostics) and resolved by SDS-PAGE. The various primary Abs used were mouse monoclonal anti-APE1 (Novus), anti-FLAG (Sigma), anti-HSC70 (catalog number B6-sc7298; Santa Cruz Biotechnology), anti-α-tubulin (catalog number T6199; Sigma), anti-AcAPE1, and anti-mouse Sin3a Ab (catalog number sc994; Santa Cruz).

### AP endonuclease activity assay.

A 43-mer oligonucleotide containing the apurinic/apyrimidinic (AP) site analog tetrahydrofuran (THF) at nucleotide 31 (Midland Corp.) was 5′ end labeled with [γ-^32^P]ATP using T4 polynucleotide kinase as described previously (54–56). Following annealing to the complementary strand with an opposite THF, the duplex oligomer was purified by use of a gel filtration column (Chroma Spin TE 10; Clontech). This THF-containing duplex oligomer was incubated with recombinant WT APE1 or recombinant AcAPE1, prepared as described earlier (15, 24), at 37°C for 3 min, during which the reaction rate in a 15-μl reaction mixture containing 50 mM Tris-HCl, pH 8.5, 50 mM KCl, 1 mM MgCl_2_, 1 mM DTT, 0.1 mM EDTA, and 100 μg/ml bovine serum albumin was linear. The reaction was stopped with 10 μl 80% formamide–40 mM NaOH containing 0.05% xylene cyanol, followed by heating at 95°C for 5 min. The samples were run in a denaturing gel in 20% polyacrylamide containing 8 M urea to separate the substrate oligomer from the cleaved product. The gels were dried, and the radioactivity was quantitated by PhosphorImager analysis in a Storm system (Molecular Dynamics). The kinetic parameters *K_m_* and *k*_cat_ were calculated by incubating 33 pM enzyme at 37°C for 3 min with substrates at various concentrations (0 to 160 nM). The enzyme kinetics data were fitted by nonlinear least-squares regression to obtain *V*_max_ and *K_m_* values by use of the Michaelis-Menten equation and SigmaPlot software.

### AP site measurement assay.

After endogenous APE1 downregulation in HEK293T^APE1siRNA^ cells with Dox treatment, the cells were transfected with WT or mutant APE1 expression constructs, as described above. At 48 h posttransfection, the cells were treated with glucose oxidase for 30 min or not treated and total genomic DNA was isolated by use of a Qiagen DNeasy kit following the manufacturer's protocol. The number of AP sites was measured using an aldehyde-reactive probe (Dojindo Laboratories) according to the manufacturer's protocol.

### UV fluorescence.

The recombinant APE1 or AcAPE1 protein solution in PBS buffer (pH 7.5) was excited at 280 nm at 25°C, and the emission was monitored at wavelengths ranging from 300 to 450 nm. The average spectrum was obtained from triplicate measurements.

### ChIP assay and ChIP followed by Western blotting.

Cross-linked chromatin was immunoprecipitated with anti-AcAPE1 antibody, and p21 promoter-directed chromatin immunoprecipitation (ChIP) analysis was carried out essentially as described previously (53). ChIP followed by Western blotting was performed as described previously (57) using anti-OGG1.

### Colony formation assay.

The generation of HEK293T^APE1siRNA^ cells, which stably expressed APE1 siRNA from a doxycycline-inducible promoter, was described earlier (15). HEK293T^APE1siRNA^ cells were treated with doxycycline (1 μg/ml; Sigma) for 5 to 6 days to knock down endogenous APE1 and transfected with expression plasmids containing WT APE1 or one of the different APE1 mutants. Equal numbers (approximately 500) of cells plated on 60-mm plates were treated with glucose oxidase (100 ng/ml for 30 min) or not treated. After the cells were washed, fresh medium was added and the cells were allowed to grow for 2 weeks until visible colonies appeared. The colonies were fixed with 100% methanol, stained with Giemsa staining solution (1:50), and counted. HCT116 cells stably expressing APE1-specific shRNA (HCT116^APE1shRNA^ cells) were transfected with WT APE1 or an APE1 mutant. At 48 h after transfection, approximately 500 cells on 60-mm dishes were treated with various doses of methyl methanesulfonate (MMS; 0.5, 1, 1.5, and 2 mM) for 1 h and then washed, fresh medium was added, and the cells were allowed to grow for 2 weeks until visible colonies appeared. HCT116 cells expressing control shRNA were used as a control.
